# Reconsidering the Usefulness of Adding Naloxone to Buprenorphine

**DOI:** 10.3389/fpsyt.2020.549272

**Published:** 2020-09-11

**Authors:** Christopher K. Blazes, Jonathan D. Morrow

**Affiliations:** ^1^ Department of Psychiatry, University of Michigan, Ann Arbor, MI, United States; ^2^ Neuroscience Program, University of Michigan, Ann Arbor, MI, United States

**Keywords:** opioids, substance use disorders, addiction, overdose, diversion, agonist therapy

## Introduction

We are in the middle of an opioid epidemic with tens of thousands of lives lost every year. As we combat this problem, it is critically important that we continually scrutinize our research efforts and care strategies in the spirit of the scientific method. Especially in light of a death toll that lowered overall US life expectancy for the first time since the flu pandemic in World War I, (1) we must maintain our readiness to reconsider well-established theories and practices in order to improve our efforts to contain this crisis. These efforts will require precision and accuracy in our translation of the literature base. One of the most effective interventions for opioid use disorder has been buprenorphine maintenance therapy, largely using a combination of buprenorphine and naloxone. However, evidence accumulated particularly over the last decade indicates that adding naloxone to buprenorphine may not be as effective a deterrent to misuse by parenteral (i.e., outside the gastrointestinal tract) administration as once thought, and for many patients, naloxone may in fact make the combination product less safe than buprenorphine monotherapy.

Buprenorphine has been used as a monotherapy product since the 1970s. Buprenorphine was combined with naloxone and released as a combination product in the United States in 2002. It was marketed as a sublingual formulation less likely to be abused and injected. This assertion was based on the fact that buprenorphine has relatively high bioavailability with sublingual absorption (35%–55%) compared to naloxone (less than 10%). When administered parenterally naloxone, which is a strong μ opioid antagonist, would be expected to block the partial μ agonist effects of buprenorphine, thereby discouraging such misuse of the product. However, if used as directed the sublingual absorption of naloxone should be less than 10% and, theoretically, not interfere with the pharmacologic actions of buprenorphine. This characterization of the combination product has been generally accepted by the medical community since buprenorphine/naloxone was FDA approved in 2002. This view is reflected in the description of buprenorphine/naloxone products provided by the Substance Abuse and Mental Health Services Administration (SAMHSA) which states, “Because of buprenorphine’s opioid effects, it can be misused, particularly by people who do not have an opioid dependency. Naloxone is added to buprenorphine to decrease the likelihood of diversion and misuse of the combination drug product. When these products are taken as sublingual tablets, buprenorphine’s opioid effects dominate naloxone and blocks opioid withdrawals. If the sublingual tablets are crushed and injected, however, the naloxone effect dominates and can bring on opioid withdrawals (2).” Based largely on this characterization, it has become the standard of care to use this combination in preference to buprenorphine monotherapy in the United States except in certain special circumstances such as pregnancy.

However, patient experience commonly stands in contrast to the prevalent view of naloxone as a strong deterrent to parenteral misuse of buprenorphine/naloxone products. Many patients with substance use disorders make use of independent, non-evidence based internet harm reduction sites such as “Bluelight” and “Erowid.” These are international, online harm-reduction communities, committed to reducing the harm associated with drug use. They host forums and blogs with discussions about patterns and modes of drug use. These sites provide detailed descriptions of pharmacokinetic and pharmacodynamic properties of the substances, and show how this knowledge can be used to maximize the clinical effects of drugs while minimizing potential side effects and withdrawal syndromes. Specific instructions are readily available on these sites for dissolving different preparations of buprenorphine/naloxone and injecting them intravenously. Following these instructions, patients typically experience moderate euphoria and report no symptoms of withdrawal. Such experiences have led to a belief in the drug-using community that the naloxone in these preparations is “inert.” We turned to the literature to assess whether there is a scientific basis for this belief, especially since stigma often leads health care professionals to subconsciously discount observations from people with substance use disorders.

### Pharmacology

There is pre-clinical evidence to support the claim that naloxone has very limited effects when buprenorphine is present. First, though naloxone can displace most opioids due to its relatively high binding affinity, buprenorphine has a 10-fold greater binding affinity for the µ opioid receptor compared to naloxone (3–5). The slow receptor dissociation kinetics of buprenorphine in conjunction with the rapid elimination kinetics of naloxone further suggests that buprenorphine would largely supplant co-administered naloxone from µ opioid receptors, thus effectively rendering naloxone inert (6). Furthermore, the half-life of naloxone is only 30–40 min. Buprenorphine has a half-life of 24–60 h with other clinical effects such as analgesia and euphoria lasting at least 6 h. Any attenuation of buprenorphine’s effects by co-administered naloxone would therefore likely be short-lived. For these reasons, a monograph commissioned by the National Institute on Drug Abuse for exploring the potential of buprenorphine for treatment of opioid dependence recommended against combining sublingual formulations of buprenorphine with naloxone: “Naltrexone, which is approved for maintenance as an oral product, is preferred to naloxone for incorporation into a sublingual buprenorphine product for takehome use. Its duration of action is significantly longer than that of naloxone, more evenly matching that of buprenorphine. Naloxone’s short duration of action means that, even if present in substantial dose in the combination, it would only delay the onset of buprenorphine’s agonist effects (7).” SAMHSA’s clinical guidelines for the use of buprenorphine also state, “Those receiving prescription buprenorphine or buprenorphine/naloxone tablets who dissolve and inject their own medication: This population would experience an agonist effect from buprenorphine but no antagonist effect from naloxone, as large doses of opioid antagonists are needed to precipitate withdrawal in buprenorphine-maintained subjects (8).”

Several clinical studies have demonstrated that parenteral administration of the combined formulation causes precipitated withdrawal symptoms in opioid-dependent subjects (9–11). However, these dramatic consequences only occur under certain specific conditions, namely in subjects who are taking a full opioid agonist such as morphine or hydromorphone and still have significant concentrations of the agonist in their circulation at the time of buprenorphine/naloxone administration. This effect is cited as the main reason naloxone is added to buprenorphine formulation, but the effect is not unique to the combination product. Because it is a high-affinity partial agonist at the µ-opioid receptor, buprenorphine itself will cause precipitated withdrawal in an opioid-dependent person who has a full opioid agonist on board. The presence or absence of naloxone makes little practical difference in this clinical scenario.

### Effects on Reward

One of the main findings leading to the conclusion that the combination product has significantly reduced abuse liability is that intravenous naloxone reduces the subjective rewarding effects of buprenorphine. For example, Jones et al. reported in 2017 that naloxone produces an “almost complete attenuation of reinforcing and positive subjective effects” of buprenorphine (12). This reduction of subjective effects has indeed been a consistent finding in multiple clinical studies (12–15), however many of those same studies also showed that the attenuation was only temporary (13, 15, 16). Most subjects report feeling a comparable “high” to buprenorphine alone just 20 to 30 min after co-injection of buprenorphine and naloxone. Though slower pharmacodynamics are known to reduce abuse liability (17), a 20- or 30-min delay in the onset of action is still more than capable of supporting addictive behavior, as evidenced by the widespread abuse of immediate-release oxycodone, whose subjective effects typically peak 1–2 h after ingestion (18).

In any case, multiple lines of evidence have suggested that the subjective effects of drugs are not the primary determinants of their abuse liability. Rather, addictive drug use is driven by a desire to pursue drug-associated rewards that is largely subconscious, sensitizes with repeated drug exposures, and can be entirely dissociated from the pleasurable effects of the drug (19–22). In fact, the pleasurable effects of drugs typically fade away as the user builds tolerance, while the desire to use only grows stronger. The effects of naloxone on actual intravenous self-administration of buprenorphine have been decidedly less clear than the subjective consequences of such use. One study found intravenous self-administration of buprenorphine/naloxone to be statistically lower than that of buprenorphine alone (13), while two other studies from the same group found no statistical difference between the two formulations (12, 15). Empirically, intravenous injection of buprenorphine/naloxone is quite common and documented in the literature (23–28). One study showed that 46% of patients on maintenance therapy (buprenorphine or methadone) have injected buprenorphine intravenously (28).

There is also a recent documented trend to misuse buprenorphine tablets through insufflation (snorting). It is commonly known that naloxone is absorbed readily through intranasal administration. This fact is exploited by the naloxone nasal spray, a single use insufflator used in opioid overdoses. Insufflation provides significantly higher bioavailability for both buprenorphine (up to 48% vs 30% sublingual) and naloxone (up to 30% vs 10% sublingual) (29). Studies of the potential effects of naloxone on the propensity to misuse of buprenorphine *via* insufflation mirror the findings on intravenous administration. Several studies have reported substantial subjective rewarding effects from insufflated buprenorphine/naloxone, and there are no statistically significant differences with regard to actual intranasal self-administration between buprenorphine alone and buprenorphine/naloxone (29, 30).

### Tolerance

Studies have shown no differences in safety or efficacy between the monotherapy product and combination formulation. However, it is not unusual for early clinical trials to overlook longer-term effects that may actually be harmful to patients. For example, one recent study comparing buprenorphine to buprenorphine/naloxone found no differences in mortality while patients were on treatment, but after treatment cessation mortality rates were significantly higher among patients who had been on the combination product (31). Preclinical studies have shown that prolonged exposure to even small amounts of µ antagonists such as naltrexone or naloxone can result in upregulation of µ receptors and a loss of tolerance for opioid-dependent individuals (32–35). These findings, in conjunction with a number of reported overdose deaths in the immediate aftermath of naltrexone treatment, have raised concerns that chronic use of opioid antagonists can predispose to fatal and non-fatal overdoses upon discontinuation of treatment (36). As noted above, oral administration of naloxone substantially reduces but does not eliminate its bioavailability. Naloxone is detectable in the urine of almost all patients taking sublingual naloxone/buprenorphine, with a median level of 60–70 ng/ml (37, 38). Tolerance to chronic opioids arises in part due to a shift in µ-opioid receptor effects from inhibitory to excitatory, and concentrations of naloxone considerably lower than 60 ng/ml are capable of reducing opioid tolerance by shifting intracellular coupling of µ-opioid receptors away from excitatory G_s_ proteins and back toward inhibitory G_i/o_ proteins (39–42). If there were clear evidence that naloxone is effective at preventing misuse of buprenorphine, then an argument could be made that these potential risks are acceptable in light of proven benefits of combination therapy. Conversely, if naloxone does not act as a deterrent to parenteral administration of buprenorphine, then exposing patients to its potentially life-threatening side effects becomes harder to justify.

## Discussion

Based on the evidence outlined above, we cannot unambiguously conclude that naloxone is an effective deterrent to parenteral misuse of buprenorphine. At best, naloxone may reduce or delay the subjective “high” users experience, but in the absence of any dramatic effect on abuse liability, this partial blockade of subjective euphoric effects is of dubious clinical value. Epidemiologic studies have documented reductions in parenteral misuse of buprenorphine after introduction of the combination product, but some of this effect this may simply be due to patients hearing from their physician or from others in the medical community that naloxone prevents such misuse. It could be argued that, if it prevents a patient from ever attempting to take a buprenorphine/naloxone product parenterally, the message that naloxone blocks such misuse is of net benefit to the patient regardless of the actual pharmacological efficacy of naloxone in this regard. However, deliberately misleading patients is an ethical violation, even if we think it is in their best interest. This is one reason that, despite their many proven benefits, we do not actually prescribe placebos. The effectiveness of such interventions depends on trust that has been painstakingly cultivated over generations of interactions between the medical community and the public we serve. If information circulating in the recreational drug-using community is in reality more accurate than the information coming from the medical community, it can only be a matter of time before that hard-won trust is eroded. Our patients expect us to be honest and straightforward with them about the risks they face, and especially about the interventions, we recommend. The stakes are too high for us to do anything less.

## Author Contributions

CB contributed the conception and wrote the first draft of the manuscript; CB and JM wrote sections of the manuscript. All authors contributed to the article and approved the submitted version.

## Conflict of Interest

The authors declare that the research was conducted in the absence of any commercial or financial relationships that could be construed as a potential conflict of interest.
